# Dr. Lippe and the I. H. A.

**Published:** 1883-12

**Authors:** C. Pearson


### DR. LIPPE AND THE I. H. A.

It is more in sorrow than in anger that we reply to the strictures of Dr. Lippe in the November number of THE PHYSICIAN in reference to the International Hahnemannian Association. It is not the intention that any word or act of mine should mar in the least degree a friendship that has long existed between us. I know the Doctor is a "good hater," and it is my misfortune, rather than my fault, that I am the same, but I do not believe there is any great diversity of opinion between us, and therefore it is only necessary to correct a misunderstanding. I would not attempt to even do this were it not that prominent members of the I. H. A. think as the executive officer of the Association it is my duty to do so. The Doctor's premises are wrong, and consequently his conclusions are far from correct. He seems in some way to have got the impression that the I. H. A. has abandoned Homœopathy and adopted the nosode theory of Dr. Swan, which he calls Isopathy, and in consequence he has no use for either Dr. Swan, the I. H. A., or for any one who may entertain adverse views from his own. It is not proposed in this connection to defend Dr. Swan—he is amply competent to do this for himself—but it is proposed to show that when Dr. L. attempts to establish what he says is an "historical fact," that the I. H. A. has by resolution or otherwise indorsed the opinions of Dr. Swan, Dr. Lippe, or any other man, except Dr. Hahnemann, he is fearfully mistaken, and has set up a man of straw, on which he has been training his artillery for over a year. But to the Doctor's language: He says, "It is singular that this International Hahnemannian Association, now in existence for over three years, has never yet made the slightest attempt to *reform* the Institute, but, on the contrary, *sanctions* the introduction by one of its members of a vastly viler heresy than the Institute ever presented, or, under the plea of freedom of medical opinion and action, allowed it to remain unnoticed. But here are historical facts to show the correctness of our assertions. Nothing has been done to vindicate the publicly expressed promises of eliminating from the Institute departures which have crept into that organization." "Publicly expressed promises." When were these promises made, and by whom? Dr. L. was himself one of the organizers of this Association. Did he make the promises, and did he suppose it would be possible for this Association to *reform* the Institute? For our part, we had no such sanguine expectations. He is himself, we believe, president of a medical society in Philadelphia, which bears his name. What has it ever done to reform the Institute? Can he enlighten us on this subject? The "Lippe Society" has been in existence longer than the I. H. A., and certainly since its organization it should have made some effort at reform, but the "Executive Committee, with the president at its head," seemed to have remained silent in this particular. He says the I. H. A. *sanctions* the dreadful heresies of which he complains. How, when, and in what way? Will he be kind enough to inform us? Has the Association ever passed any resolution to this effect? Has any such resolution ever been proposed? Has the president, or any of the officers, ever said or published one word looking in this direction? If so, the history of the Association, with which Dr. L. seems to be so familiar, certainly fails to show it. But the Doctor says, it has done this by allowing the heresy "to remain unnoticed." Now, this is much like the boy's composition on pins; he said pins had saved the lives of a great many children by their not having swallowed them. Dr. Swan has never attempted to force his views on the Association, and we are of the opinion that had Dr. Lippe adopted the policy of the I. H. A. and remained silent in reference to them they would have attained much less notoriety. The danger is not always in proportion to the noise that cats make; they are sometimes simply making more cats; and the more noise made about nosodes, and the more the subject is agitated, the more likely it is to be investigated, and perhaps the more friends it will gain. Besides, the I. H. A. is an independent body, and has little to do with the individual opinions of members who have pledged themselves to abide by the platform of principles adopted when the Association was first organized; it is not disposed to "quarrel with a man because he has one hair more in his beard" than the rest of us, but we propose to let him alone unless he undertakes to trim all our beards to his standard, then it will be time enough to adopt resolutions. Dr. Gregg, a most excellent physician, an active member, and the present vice-president of the Association, entertains original ideas in regard to the germ origin of disease. These views are his own; he has a perfect right to them; he has never attempted to obtrude them on other members of the Association, though there are members heartily in accord with him, and if others may not be, it makes no difference so far as the Association is concerned. We like to see men think and investigate. Any one who never changes his opinions never corrects mistakes, and yet it is preposterous to assert that the Association has sanctioned the opinions of Dr. Gregg by remaining silent, or that it can in any way be held responsible for them.

But, says Dr. L., this is a different thing. Yes, it is the difference between the cause and cure of disease, the difference between a nosode and a germ, but the principle nevertheless is the same. The Doctor says it is an "historical fact" that these heresies, as he is pleased to call them, are "sustained" by the officers and Executive Committee of the I. H. A.

Yes, sustained just as we have shown, because we have not seen proper to follow the lead of Dr. Lippe and censure Dr. Swan. If Dr. L. had grievances to correct, or supposed that these heresies were growing in the Association, why did he absent himself from its meetings and complain of what was done, or rather what was not done? Why did he not attend in person and use his influence, and give his counsel that the Association might not be tainted by making and worshiping in his absence a golden calf? It is, however, an "historical fact," that at no meeting of the Association at which Dr. L. had not been present, has one paper been read, one suggestion been made, or one *word said* in discussion to which Samuel Hahnemann himself had he been present would have taken exceptions. Again, it comes with bad grace from one who prescribes the hundred thousandth potency, to stigmatize others in the slang phrase of the mongrels as the "bottle-washers." But perhaps the Doctor thinks all weapons are lawful in war; he seems to think that some one entertains the belief that a non-homœopathic agent, "even a nosode, a supposed product of a disease, becomes a truly homœopathic remedy for the disease itself by submitting it to a process of potentization." Just to whom this charge is intended to apply is not made very clear, perhaps, to Samuel Hahnemann, for he said: I call psorin a *homœopathic* anti-psoric, because if the preparations of psorin did not alter its nature to that of a homœopathic remedy, it never could have any effect upon an organism tainted with that same identical virus. The psoric virus, by undergoing the processes of trituration and shaking, becomes just as much altered in its nature as gold does, the homœopathic preparations of which are not inert substances in the animal economy, but powerful acting agents. Psorin is a *simillimum* of the itch virus. There is no intermediate degree between *idem* and *simillimum;* in other words, the thinking man sees that simillimum is the medium between simile and idem. The only definite meaning which the terms "isopathic and æquale" can convey is that of simillimum; they are not *idem.* (Vide *Chronic Diseases*, volume I, page 196.) Will Dr. Lippe please settle this matter with Dr. Hahnemann in his forthcoming paper on the "Past, Present, and Future of Homœopathy"?

C. PEARSON.

